# Effect of melatonin on sleep quality and EEG features in childhood epilepsy: a possible non-conventional treatment

**DOI:** 10.3389/fneur.2023.1243917

**Published:** 2023-09-14

**Authors:** Giovanni Battista Dell'Isola, Giorgia Tascini, Valerio Vinti, Eleonora Tulli, Gianluca Dini, Elisabetta Mencaroni, Pietro Ferrara, Giuseppe Di Cara, Pasquale Striano, Alberto Verrotti

**Affiliations:** ^1^Department of Pediatrics, University of Perugia, Perugia, Italy; ^2^Unit of Pediatrics, Città di Castello Hospital, Città di Castello, Italy; ^3^Unit of Pediatrics, Campus Bio-Medico University, Rome, Italy; ^4^Department of Neurosciences, Rehabilitation, Ophthalmology, Genetics, Maternal and Child Health, University of Genoa, Genoa, Italy

**Keywords:** epilepsy, melatonin, sleep disorders, antiseizure medication, EEG

## Abstract

**Background:**

Sleep and epilepsy are characterized by a bidirectional relationship. Indeed, epilepsy predisposes to the development of sleep disorders, while sleep deprivation may exacerbate epilepsy. In addition, antiseizure medication can disrupt normal sleep architecture. Therefore, adequate sleep hygiene could lead to improvement in seizure control. The present study aimed to evaluate the effect of melatonin on seizure frequency, EEG tracing, and sleep in children with focal idiopathic epilepsy.

**Methods:**

This observation study evaluated the effect of 4 mg oral melatonin in ameliorating sleep–wake cycle, seizure frequency, and EEG features in children with focal idiopathic epilepsy of infancy. Twenty children were enrolled from September 2020 to August 2021. The study consisted of serial controls at enrollment (t0), at 3 months (t1), and at 6 months (t2) including neurological examination, questionnaire about sleep disturbances (CSHQ), and EEG.

**Results:**

A significant improvement in sleep quality and daytime sleepiness was observed after melatonin supplementation. Furthermore, we observed a noteworthy improvement in EEG tracing at t2 that exhibited a significant correlation with improvements in CSHQ scores.

**Conclusion:**

The studies conducted so far to evaluate the effect of melatonin in persons with epilepsy do not lead to definitive conclusions. Despite the small population sample and the study design, we report sleep and EEG improvement after melatonin administration in our cohort. Larger studies are needed to further study the neuroprotective and anticonvulsant properties of melatonin.

## Introduction

Melatonin (N-acetyl-5-methoxytryptamine) is an indole hormone produced from tryptophan in the pineal gland according to the circadian rhythm (1). Melatonin secretion begins in the early evening, peaking between 02:00 and 04:00 a.m., and its half-life is approximately 45 min (2).

Melatonin regulates circadian rhythms such as sleep–wake rhythms, neuroendocrine rhythms, and body temperature cycles and also acts in many peripheral tissues such as the pancreas, liver, kidney, heart, lung, adipose, and intestine (3–6).

### Epilepsy and melatonin

There are contradictory data regarding melatonin levels in persons with epilepsy. Molina-Carballo (7) observed high levels of melatonin after seizures, while in other studies, low levels of melatonin were reported in persons with epilepsy (8, 9). In a study conducted on 91 children and adolescents with epilepsy aged between 9 months and 18 years, blood levels of melatonin were measured 30 min and 24 h after a seizure (10). Basal melatonin levels were found to be lower in children with seizures compared with healthy controls. However, some authors reported increased melatonin levels during seizures. Dabak et al. compared serum melatonin levels measured in the 1st h and also 12 and 24 h after seizure, reporting significantly elevated levels in the 1st h (11). Mahyar et al. reported no significant difference in serum melatonin levels after seizure in epileptic children vs. febrile children without seizures (12). However, melatonin dosage was performed over a 24-h period, which is very long considering a melatonin half-life of almost 30 min.

Melatonin has shown anticonvulsant properties in several studies conducted in animal models (13–16). For example, intraventricularly injections of antimelatonin antibodies caused transitory epileptiform abnormalities in rats (17). Confirming this hypothesis, regular injections of melatonin in gerbils led to a significant decrease in both the severity and frequency of chemically induced seizures (18).

Gupta et al. demonstrated in murine models an increased efficacy of phenytoin and carbamazepine when used in combination with melatonin (19). The favorable results emerged from studies on animal models and encouraged the use of melatonin for seizure control in humans.

### Melatonin effect on persons with epilepsy

There is conflicting evidence regarding melatonin's efficacy in reducing seizures. While some authors described melatonin's role as an anticonvulsant and neuronal protector (20), others reported a proconvulsant effect of melatonin (21, 22).

In a randomized, controlled clinical trial, 25 patients (aged from 3.6 to 26 years) all affected by mental disabilities, mostly with seizures, were randomized to receive oral melatonin vs. placebo. Melatonin was effective in improving sleep disorders and had less efficacy on epilepsy (23).

In a double-blind, randomized trial conducted on 11 children, the effect of melatonin (9 mg/day for 4 weeks) on sleep and seizures was evaluated. The results showed a significant reduction in sleep latency and wakefulness after sleep onset. No clear effects on seizures have been highlighted probably due to the small number of patients (24). In a study conducted on 10 children and adults with refractory epilepsy, the treatment with 10 mg/day of melatonin decreased diurnal seizures (25). Ross et al. evaluated the efficacy of melatonin in 49 children with neurodevelopmental disorders. The study showed a reduction in sleep disturbances in 34 patients. In addition, among 26/49 enrolled children presenting with epilepsy, 3 reported an improvement in seizure control (26). In a small trial, treatment with 3 mg of oral melatonin 30 min before bedtime reduced seizures in 5 out of 6 children with refractory epilepsy (27). In 2011, Uberos et al. reported better control of seizures in children with severe epileptic disorders after treatment with melatonin (28). Fauteck et al. administered melatonin to 10 children with severe neurological impairment (6 affected by Lennox-Gastaut syndrome) (29). In six children, a single evening dose of 5–10 mg of melatonin resulted in a clear improvement in seizure control. A single case has been reported using higher doses of melatonin (up to 500 mg/day) in a child with severe myoclonic epilepsy with beneficial effects on seizure control (30).

In a 2019 randomized clinical trial, 60 children (aged six to 50 months) with recurrent febrile seizures were randomized to receive oral melatonin 0.3 mg/kg/8 h (*n* = 30) or oral diazepam 1 mg/kg/day, divided into three doses (*n* = 30). Both melatonin and diazepam were given only during febrile illness, and patients were followed up for 6 months. Both melatonin and diazepam significantly reduced the recurrence of febrile seizures without significant differences between the two groups (31).

In contrast with the reported data, other authors found that melatonin may increase epileptic activity in humans. In a study conducted by Sheldon, melatonin showed proconvulsant effects in a group of six children with multiple neurological deficits (21). In a retrospective study on 13 children and young adults with severe learning disabilities and behavioral disorders, treatment with melatonin resulted in increased seizures in three patients and decreased seizures in seven patients (32). In 2005, Hancock evaluated the efficacy of add-on melatonin (5 or 10 mg) in eight children and adults (aged 18 months to 31 years) with tuberous sclerosis and epilepsy. The study found no benefit in seizure control for either dose of melatonin (33).

The present study aimed to evaluate the effect of melatonin on seizure frequency, EEG tracing, and sleep in children with focal idiopathic epilepsy.

## Methods

### Study design

The present study was an 18-month observational prospective study, conducted in the Pediatric Neurology outpatient service of the University Hospital of Perugia. The registry started in September 2020, and patients were monitored for 6 months. All patients' legal guardians provided written informed consent before participation. The study was conducted in accordance with the Declaration of Helsinki, and all parents provided written informed consent for the study.

### Study procedure and data collection

Children with epilepsy attending the Pediatric Neurology Outpatient Service who fulfilled the following inclusion criteria were enrolled from September 2020 to August 2021.

The inclusion criteria are as follows:

- Children with focal idiopathic epilepsy, aged between 2 and 18 years of either gender, taking or not antiepileptic drugs. The diagnosis of focal idiopathic epilepsy was based on history, age at onset, and electroclinical and neuroimaging findings.
- Children who underwent a regular neurological follow-up.

The exclusion criteria are as follows:

- Children diagnosed with other forms of epilepsy.
- Neuroradiological abnormalities.

Children and adolescents with epilepsy attending the Pediatric Neurology Outpatient Service of the University Hospital of Perugia were screened and enrolled according to the selection criteria. After recruitment, detailed medical history, physical, neurological, and psychiatric examinations were performed.

In children under antiseizure medication (ASM), the drug serum level was tested at enrollment and 6 months. Patients were administered 4 mg a day of melatonin, 30–40 min before bedtime, from the day of enrollment to the following 6 months. We have chosen the dose of melatonin as 4 mg daily which is within the therapeutic dose range of melatonin (2–5 mg) according to Vecchierini et al. (34).

The study consisted of serial controls at enrollment (t0), at 3 months (t1), and at 6 months (t2) to monitor the following data:

- Neurological examination at t0 and t2.
- Questionnaire about sleep disturbances (CSHQ questionnaire) at t0, t1, and t2.
- EEG monitoring at t0 and t2.

In agreement with those criteria, we have studied 20 children and adolescents in the study, of which 16 were male and 4 female patients. One patient was excluded for poor adherence to therapy. The cohort involved was aged between 5.1 and 16.3 years old with an average of 10.2 years. All were affected by idiopathic focal epilepsy with a mean age at seizure onset of 7.47 years. Based on the ILAE 2017 criteria for seizure (35), 14 (73.7%) children presented generalized seizure at onset, of which 3 had a status epilepticus, and 5 children presented focal seizure at onset. In total, 13 children had a diagnosis of self-limited epilepsy with centrotemporal spikes (SeLECTS, 11 males and 2 females), 3 had temporo-occipital epilepsy (2 male and 1 female patients), 1 had occipital epilepsy, 1 had frontal epilepsy, and 1 had temporal epilepsy (Table 1). Eight of the 20 children enrolled were treated with an ASM: 6 with carbamazepine and 2 with valproate. All enrolled patients presented with normal brain MRI. Psychiatric examination was normal in all recruited children.

**Table 1:** Baseline clinical and demographic characteristics.

**Parameters**	**Population**
Total number	19
Mean age (years)	10.2
Male/female ratio	16:3
Age at onset (years)	7.47
Duration of epilepsy (years)	2.5
**Types of epilepsy:**	
– SeLECTS	13 (69.4%)
– Temporal epilepsy	1 (5.2%)
– Temporo-occipital epilepsy	3 (16%)
– Occipital epilepsy	1 (5.2%)
– Frontal epilepsy	1 (5.2%)
**Seizure onset:**	
– Focal	5 (26%)
– Generalized	14 (74%)
**ASMs**	8 (42%)

### Study outcome

Our primary outcome was to verify whether oral melatonin, both as an add-on therapy to ASM or not, could have a beneficial effect in the reduction of seizure frequency. In addition, we estimated the improvement of EEG features in patients taking ASMs or not during regular treatment with melatonin. Each patient underwent a video-EEG performed using the 10–20 international system. EEG results were evaluated by a blinded examiner. EEG improvement was assessed by analysis of background activity, presence/absence of physiological elements (especially during sleep), and interictal discharges.

A secondary aim of the study was to investigate the efficacy and safety of melatonin in ameliorating sleep–wake cycle. We assessed the child's inability to go to bed, the delay in falling asleep, sleep duration, overnight awakenings, anxiety related to sleep, parasomnia, respiratory disorders, and daytime sleepiness. The improvement of sleep quality during regular treatment with melatonin was tested through the Children's Sleep Habits Questionnaire (CSHQ) (36). CSHQ is a retrospective questionnaire in which caregivers describe their child's sleep habits for 1 week by answering 33 questions (score between 1 and 3). The higher the score, the higher the risk of sleep disturbances.

Finally, we evaluated the correlation between the CSHQ and the EEG results at t0 and t2 through the application of an independent sample t-test.

### Statistical analysis

For determining the significance of the changes between t0, t1, and t2 of each individual variable considered, given the small sample number, in order to minimize the effects of a possible non-normal distribution, the Wilcoxon test for paired data was used. A difference with a *p* < 0.05 was considered to be statistically significant. Statistical analysis was performed using SPSS 20.0 software for Windows.

## Results

### Change in seizure frequency

All children showed good tolerance to melatonin administered at 4 mg/day in the absence of side effects. At t0, five children (three with SeLECTS, one with temporo-occipital epilepsy, and one with temporal epilepsy) presented seizures within the previous 6 months. In particular, four children presented only one seizure and one patient presented three seizures with a frequency of one episode per month in the last 3 months before enrollment. All showed generalized seizures, and no one had epileptic status. At t1, only one of these children presented an additional critical episode with similar characteristics and did not report further seizures at t2. At t2, only one child with SeLECTS presented three episodes, while the others remained seizure-free.

The remaining 11 children presented no critical episodes in the 6 months prior to enrollment. After melatonin administration, all remained seizure-free, with the exception of only one patient who manifested two critical episodes at t2. Seizure frequencies at t0, t1, and t2 are reported in Table 2.

**Table 2:** Seizure frequency (SF) T0: seizures within the previous 6 months; SF T1: 3-mount seizure outcome; SF T2: 6-mount seizure outcome.

**Patient**	**SF T0**	**SF T1**	**SF T2**	**EEG T0**	**EEG T2**
1	-	-	-	Pathological	Unchanged
2	-	-	-	Pathological	Unchanged
3	-	-	-	Pathological	Unchanged
4	1	1	-	Pathological	Unchanged
5	3	-	-	Pathological	Normalized
6	1	-	-	Pathological	Normalized
7	0	-	2	Pathological	Unchanged
8	1	-	3	Pathological	Normalized
9	-	-	-	Pathological	Unchanged
10	-	-	-	Pathological	Normalized
11	-	-	-	Pathological	Normalized
12	1	-	-	Pathological	Improved
13	-	-	-	Pathological	Improved
14	-	-	-	Pathological	Unchanged
15	-	-	-	Pathological	Improved
16	-	-	-	Pathological	Improved
17	-	-	-	Normal	Unchanged
18	-	-	-	Pathological	Unchanged
19	-	-	-	Pathological	Unchanged

Improved EEG at t2 was defined as a reduction of more than 50% of interictal paroxysmal activity.

However, statistical analysis did not show an improvement in seizure frequency after melatonin administration.

### Change in EEG features

EEG performed at t0 showed paroxysmal interictal activity in 18 children (94%). At t2, five children (two with SeLECTS, one with occipital epilepsy, one with temporo-occipital epilepsy, and one with temporal epilepsy) presented a normalization of EEG, and four children showed a substantial reduction in paroxysmal interictal activity (defined as >50% activity). In 50% of patients with an active interictal EEG at t0, there was no evidence of improvement at t2.

Statistical analysis showed a significant improvement in the EEG at t2 (*p*-value: 0.025). EEG improvement was independent of both patients' age at enrollment and therapy. In addition, patients on antiepileptic therapy showed no changes in ASM plasma concentration during the co-administration of melatonin. Finally, EEG improvement was significantly correlated with sleep improvement (*p*-value: 0.03).

### Change in sleep quality and daytime sleepiness (CSHQ: children's sleep habits questionnaire)

From the comparison of CHSQ results at t0, t1, and t2, an improvement after melatonin therapy was observed. The results showed an average value of 50.3 at t0. Lower overall scores were observed at t1 and t2, with an average of 41.7 and 44.3, respectively.

Statistical analysis showed a significant reduction in the CSHQ overall value between t0 and t1 (*p*-value = 0.002) and between t0 and t2 (*p*-value = 0.01).

Furthermore, CHSQ items have been split into three subgroups:

A. Bedtime resistance, sleep onset delay, and sleep duration
B. Daytime sleepiness
C. Sleep anxiety, night wakings, parasomnias, and sleep-disordered breathing.

Comparison of individual groups at t0, t1, and t2 also showed a significant improvement in groups A and C. In contrast, the improvement obtained in subgroup B after melatonin therapy was not statistically significant.

## Discussion

Our study demonstrates a significant electroencephalographic improvement after 6 months of melatonin administration. Indeed, five children (26%) showed complete normalization of EEG tracing, while four children (21%) showed a significant improvement in interictal EEG. Similarly, sleep improvement was observed at t1 and t2. EEG improvement was significantly correlated with CHSQ results. Thus, given the melatonin nature and its known beneficial effect on sleep, EEG improvement could be attributed to melatonin therapy, assuming its anticonvulsant and neuroprotective properties. However, it is difficult to attribute EEG improvement to melatonin therapy alone since idiopathic epilepsy of childhood is often characterized by a few sporadic critical episodes which typically tend to improve in the next years, even in the absence of a specific treatment (37). Thus, more extensive studies are needed to confirm these results. The close relationship between epilepsy and sleep has long been known. Indeed, epilepsy predisposes to the development of sleep disorders, while sleep deprivation may exacerbate epilepsy (38–40). In addition, ASMs can deeply modify both sleep architecture and sleep–wake cycle. Indeed, attention deficit and daytime drowsiness are common adverse effects of ASMs (41). It is also known that idiopathic epilepsy in childhood has a strong correlation with the sleep–wake cycle. In fact, affected patients present a higher concentration of critical episodes upon awakening and in the falling asleep phase. Thus, in light of its beneficial effects on sleep, safety, and ability to cross the blood–brain barrier, melatonin may have the potential to improve the quality of life of children with epilepsy. In fact, in addition to the effect on the sleep–wake cycle, melatonin's anticonvulsant properties may be related to several mechanisms (42). Indeed, it is known that melatonin exerts effects on both GABA and glutamate receptors (7) and acts on Na^+^ and K^+^-ATPase of the brain and the voltage-dependent calcium channel modulating membrane permeability and suppressing neuronal activity (7, 43). Melatonin exerts direct detoxification of radicals by restricting the activity of nitric oxide synthase (44). In addition, our study, according to previous clinical and preclinical studies (19–45), confirmed that the co-administration of melatonin with ASMs is not associated with any change in ASM plasma concentrations.

To date, the studies evaluating the effect of melatonin on persons with epilepsy do not allow us to draw definitive conclusions, often due to poor methodological quality (46). Despite the small population sample and the study design, we have witnessed a significant improvement in EEG tracing and sleep quality of children on melatonin therapy. We also demonstrated how the parent's perception of the child's sleep quality improved. Our study has some limitations related to the small sample of the patients and the absence of a control arm. However, our results are encouraging to refine further randomized trials aimed at an in-depth study of the correlation between melatonin and epilepsies.

In conclusion, according to our experience, melatonin should be considered in the treatment of idiopathic epilepsy of childhood, both in patients under ASM or not, especially in children with sleep disorders.

## Conclusion

To the best of our knowledge, this is the first pediatric study evaluating the effect of melatonin on focal idiopathic epilepsy of childhood. The beneficial effect of melatonin on the sleep–wake cycle and epilepsy has been studied so far. Currently, there are no clear indications for the use of melatonin in these patients. The limitations of our study do not allow us to draw definitive conclusions. However, melatonin, through its sleep-modulating effect, appears effective in improving EEG tracing in children with idiopathic epilepsy of childhood and probably in reducing seizure frequency. In addition, according to our experience, melatonin administered at 4 mg/day did not show side effects, confirming to be manageable and not harmful. Further prospective randomized studies are needed to draw firm conclusions, but we suggest the use of melatonin in cases of idiopathic epilepsy of childhood, both on and off ASM.

## Data availability statement

The original contributions presented in the study are included in the article/supplementary material, further inquiries can be directed to the corresponding author.

## Ethics statement

Ethical approval was not required for the study involving human samples in accordance with the local legislation and institutional requirements because the study is of negligible risk and uses only non-identifiable data about human beings. Written informed consent for participation in this study was provided by the participants' legal guardians/next of kin. Written informed consent was obtained from the individual(s) for the publication of any potentially identifiable images or data included in this article.

## Author contributions

GBD, GT, VV, ET, and GD drafted the manuscript. GBD, VV, and EM edited and revised the manuscript. EM and GT designed the data collection, performed the tests, and collected and interpreted data. GDC performed the data analysis. AV, PF, and EM supervised the manuscript and critically reviewed it. All authors contributed to the article and approved the submitted version.
